# COVID-19 Epidemic in Malaysia: Epidemic Progression, Challenges, and Response

**DOI:** 10.3389/fpubh.2021.560592

**Published:** 2021-05-07

**Authors:** Jamal Hisham Hashim, Mohammad Adam Adman, Zailina Hashim, Mohd Firdaus Mohd Radi, Soo Chen Kwan

**Affiliations:** ^1^Department of Environmental Health, Faculty of Health Sciences, Universiti Selangor, Shah Alam, Malaysia; ^2^Provenue Corporation Sdn Bhd, Subang Jaya, Malaysia; ^3^Faculty of Civil Engineering Technology, Universiti Malaysia Pahang, Gambang, Malaysia; ^4^Department of Environmental and Occupational Health, Faculty of Medicine and Health Sciences, Universiti Putra Malaysia, Serdang, Malaysia; ^5^Cheras District Health Office, Ministry of Health, Kuala Lumpur, Malaysia; ^6^Kedah State Health Department, Ministry of Health, Kedah, Malaysia; ^7^Center for Toxicology and Health Risk Research (CORE), Faculty of Health Sciences, Universiti Kebangsaan Malaysia, Kuala Lumpur, Malaysia

**Keywords:** coronavirus, pandemic, movement control order, Malaysia, COVID-19

## Abstract

COVID-19 pandemic is the greatest communicable disease outbreak to have hit Malaysia since the 1918 Spanish Flu which killed 34,644 people or 1% of the population of the then British Malaya. In 1999, the Nipah virus outbreak killed 105 Malaysians, while the SARS outbreak of 2003 claimed only 2 lives. The ongoing COVID-19 pandemic has so far claimed over 100 Malaysian lives. There were two waves of the COVID-19 cases in Malaysia. First wave of 22 cases occurred from January 25 to February 15 with no death and full recovery of all cases. The ongoing second wave, which commenced on February 27, presented cases in several clusters, the biggest of which was the Sri Petaling Tabligh cluster with an infection rate of 6.5%, and making up 47% of all cases in Malaysia. Subsequently, other clusters appeared from local mass gatherings and imported cases of Malaysians returning from overseas. Healthcare workers carry high risks of infection due to the daily exposure and management of COVID-19 in the hospitals. However, 70% of them were infected through community transmission and not while handling patients. In vulnerable groups, the incidence of COVID-19 cases was highest among the age group 55 to 64 years. In terms of fatalities, 63% were reported to be aged above 60 years, and 81% had chronic comorbidities such as diabetes, hypertension, and heart diseases. The predominant COVID-19 strain in Malaysia is strain B, which is found exclusively in East Asia. However, strain A, which is mostly found in the USA and Australia, and strain C in Europe were also present. To contain the epidemic, Malaysia implemented a Movement Control Order (MCO) beginning on March 18 in 4 phases over 2 months, ending on May 12. In terms of economic impacts, Malaysia lost RM2.4 billion a day during the MCO period, with an accumulated loss of RM63 billion up to the end of April. Since May 4, Malaysia has relaxed the MCO and opened up its economic sector to relieve its economic burden. Currently, the best approach to achieving herd immunity to COVID-19 is through vaccination rather than by acquiring it naturally. There are at least two candidate vaccines which have reached the final stage of human clinical trials. Malaysia's COVID-19 case fatality rate is lower than what it is globally; this is due to the successful implementation of early preparedness and planning, the public health and hospital system, comprehensive contact tracing, active case detection, and a strict enhanced MCO.

## Introduction

### Pandemics of the 20–21st century

Throughout the 20th century, three influenza pandemics occurred over several decades; the most severe was the “Spanish Flu” (caused by an A(H1N1) virus), estimated to have resulted in 20–50 million deaths in 1918–1919. Milder pandemics occurred subsequently in 1957–1958 (the “Asian Flu” caused by an A(H2N2) virus) and in 1968 (the “Hong Kong Flu” caused by an A(H3N2) virus), which were estimated to have caused 1–4 million deaths each.

An influenza pandemic caused by the A(H1N1) virus erupted in the 21st century (2009–2010). For the first time, a pandemic vaccine was developed, produced, and deployed in multiple countries during the first year of the pandemic. The H1N1 pandemic was however milder than the ones before, estimated to cause between 100,000 and 400,000 deaths globally in its first year (1).

### History of Epidemics in Malaysia

Newspapers in Malaya had as early as September 1918 carried reports of the raging influenza pandemic in South Asia. The only details of the spread of the epidemic were substantially documented from the medical report of the British North Borneo from June to November. The account indicates most possibly the transmission of the influenza virus from the maritime and land routes ferrying passengers and migrant workers from the South China Sea to the rest of the hinterland. This was the Spanish Flu brought in from Europe which resulted in 34,644 deaths among the 3,584,761 population then, giving a fatality rate of almost 1% (2).

Over a period of 8 months in 1999, the Nipah virus infected 265 Malaysians and killed 105. Malaysia's response was delayed because it was initially misidentified as Japanese encephalitis. The SARS outbreak of 2003, which infected 8,098 and killed 774 people globally, claimed only two lives in Malaysia. The present COVID-19 was brought into Malaysia by Chinese tourists from Wuhan *via* Singapore and Malaysian citizens who traveled to high COVID-19–infected countries such as Italy and Indonesia.

### Origin of COVID-19

Pneumonia of unknown etiology was detected in Wuhan City, Hubei Province of China on December 31, 2019, whereby, the WHO China Country Office was informed. From December 31, 2019 through January 3, 2020, a total of 44 cases of pneumonia of unknown etiology were reported in China, of which the causal agent was not identified.

On January 7, 2020, the Chinese authorities identified a new type of coronavirus. China shared the genetic sequence of the novel coronavirus for countries to use in developing specific diagnostic kits on January 12, 2020. WHO later received further detailed information from the National Health Commission China on January 11–12, 2020 that the outbreak was associated with exposures in a seafood market in Wuhan City.

On January 13, 2020, the Ministry of Public Health, Thailand reported the first imported case of laboratory-confirmed novel coronavirus case (2019-nCoV) from Wuhan, Hubei Province, China. On January 15, 2020, the Ministry of Health, Labor and Welfare, Japan reported an imported 2019-nCoV from Wuhan.

As of January 20, 2020, a total of 282 confirmed cases of 2019-nCoV have been reported in China (278 cases), Thailand (2 cases), Japan (1 case), and Korea (1 case). Cases in Thailand, Japan, and Korea were exported from Wuhan City, China. Among the 278 confirmed cases in China, 258 cases were reported from Hubei Province, 14 from Guangdong Province, 5 from Beijing Municipality, and 1 from Shanghai Municipality.

On January 30, 2020, WHO declared the outbreak of COVID-19 a public health emergency of international concern. WHO's greatest concern was the potential for the virus to spread to countries with weaker health systems which will be ill-prepared to deal with the outbreak (3).

A modeling study published in *The Lancet* on January 31 estimated that, on average, every infected individual is infecting 2.68 additional individuals (4). The specifics of how the virus is transmitted from person to person have also yet to be defined. It is still unknown whether the virus can be spread by the fecal–oral route, for example. The disease pathogenesis is shrouded in mystery. How does the virus replicate in different sites, and how does that relate to the severity of disease?

It was also uncertain how long patients remain infectious. Thus, it is difficult to decide on the period of isolation. There is also the possibility that the virus is mutating into transmissible forms. Older patients with comorbidities seem to be most at risk of developing severe disease as a result of infection with severe acute respiratory syndrome coronavirus 2 (SARS-CoV-2). Information from China stated that case fatality is around 2%.

### Transmission of COVID-19

COVID-19 is largely spread *via* droplets in the air and is a respiratory illness. These droplets are typically expelled when an infected person coughs or sneezes. They become increasingly less infectious once symptoms develop, so that a person's viral load declines steadily. However, infected persons keep shedding the virus after they recover from COVID-19 for around 2 weeks in both their saliva and stools. Infected persons with mild or no symptom can have a very high viral load in their upper respiratory tracts. They can shed the virus through spitting, touching their mouths, or noses or possibly through talking. SARS-CoV-2 has also been found to persist for days on surfaces (5).

**Figure 1:**
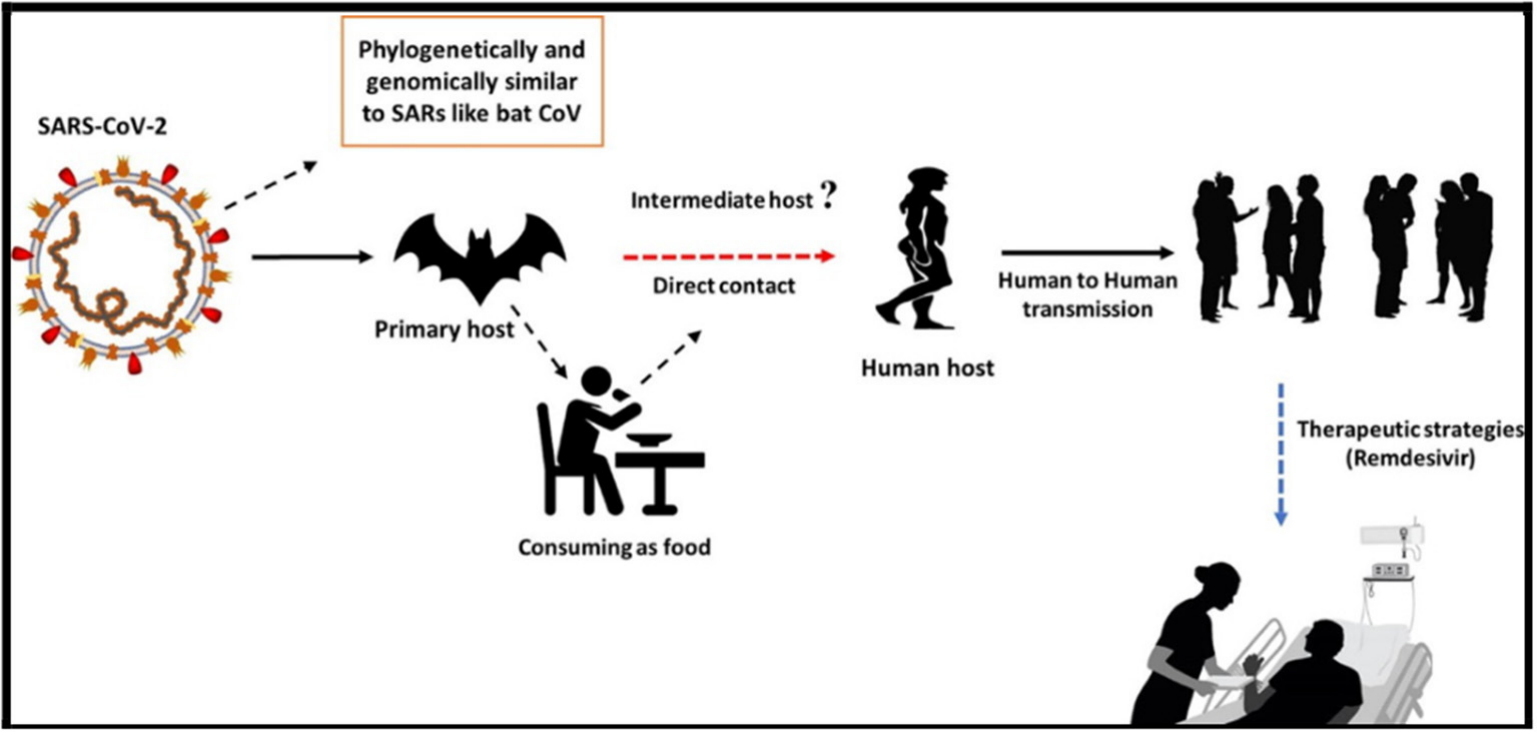
The transmission of COVID-19 (6). License: CC BY-NC-ND 4.0.

Fever, dry cough, and tiredness are the most commonly reported symptoms, and in mild cases people may get just a runny nose or a sore throat. In the most severe cases, infected persons experience breathing difficulty, and ultimately organ failure may develop. Some cases are fatal. The authorities in China have placed the Wuhan population under quarantine or lockdown, and stopped trains and flights out of the city. They have suspended certain long-distance bus routes, including those that depart or arrive in Beijing. On March 11, WHO announced the outbreak to be a pandemic, which means that multiple countries are seeing sustained transmission between people, causing disease or death (Figure 1) (6).

## COVID-19 Epidemic Progression in Malaysia

### First Wave of Outbreak (January 25 to February 15, 22 Cases)

The first three cases of COVID-19 in Malaysia were imported cases, confirmed on January 25, 2020. Imported cases are defined as infection acquired from outside Malaysia with reference to the travel history of the individual case. The three cases were detected on tracing and screening after communication from the Singapore Ministry of Health that eight close contacts of a confirmed case of a Chinese nationality in Singapore had traveled into Johor, Malaysia. By February 15, the number of cases in Malaysia increased to 22, consisting of 12 persons under investigation (PUI), eight close contacts of confirmed cases, and two Malaysian evacuees of humanitarian aid mission from Wuhan, China. Most of the cases in the first wave were imported cases or of Chinese nationality (15 out of 22 cases) and close contacts, while only two cases were of local transmission (7). After the 22nd case, no new case was reported for 11 days, which formed the first wave of COVID-19 outbreak in the country. All cases of the first wave recovered from the infection.

The *Guidelines for COVID-19 Management in Malaysia (No. 05/2020)* was developed by the Ministry of Health Malaysia in response to the novel virus (8). The guidelines define PUI as those who “developed acute respiratory infection, and had traveled/resided in foreign countries, or being in close contact with confirmed cases within 14 days before onset, or attended an event associated with a known outbreak.” Close contacts are described as those who “worked, traveled or lived together with a COVID-19 patient.”

### Second Wave of Outbreak (February 27 Onwards, 5,945 Cases by April 29)

The second wave of the outbreak started on February 27, and lasting until the present work was undertaken. From February 27, new cases began to appear as people who had international travel history to countries such as China, Japan, Italy, and Australia started to manifest symptoms. Clusters of cases began to form from the close contacts of confirmed cases who attended meetings and events together, which generated several generations of infections. The number of cases reached a total of 129 on March 10 from among the PUIs, close contacts, and evacuees of humanitarian aid missions.

### The Sri Petaling Tabligh Cluster

On March 11, a sporadic COVID-19–positive case was detected among 600 surveillance sampling of patients with influenza-like illness (ILI) and severe acute respiratory infection (SARI). At the same time, the International Health Regulations (IHR) Focal Point (FP) in Brunei informed the IHR FP Malaysia that a COVID-19 case in Brunei had attended a tabligh (Islamic missionary) convention held at Masjid Jamek in Sri Petaling, Kuala Lumpur from February 27 to March 3, 2020. The event was purportedly attended by 14,500 Malaysians who had since gone back to their respective states throughout Malaysia (9). There were also 1,500 oversea attendees who had returned to their countries across Asia. The initially sporadic case was also linked to the tabligh convention in Sri Petaling.

**Figure 2:**
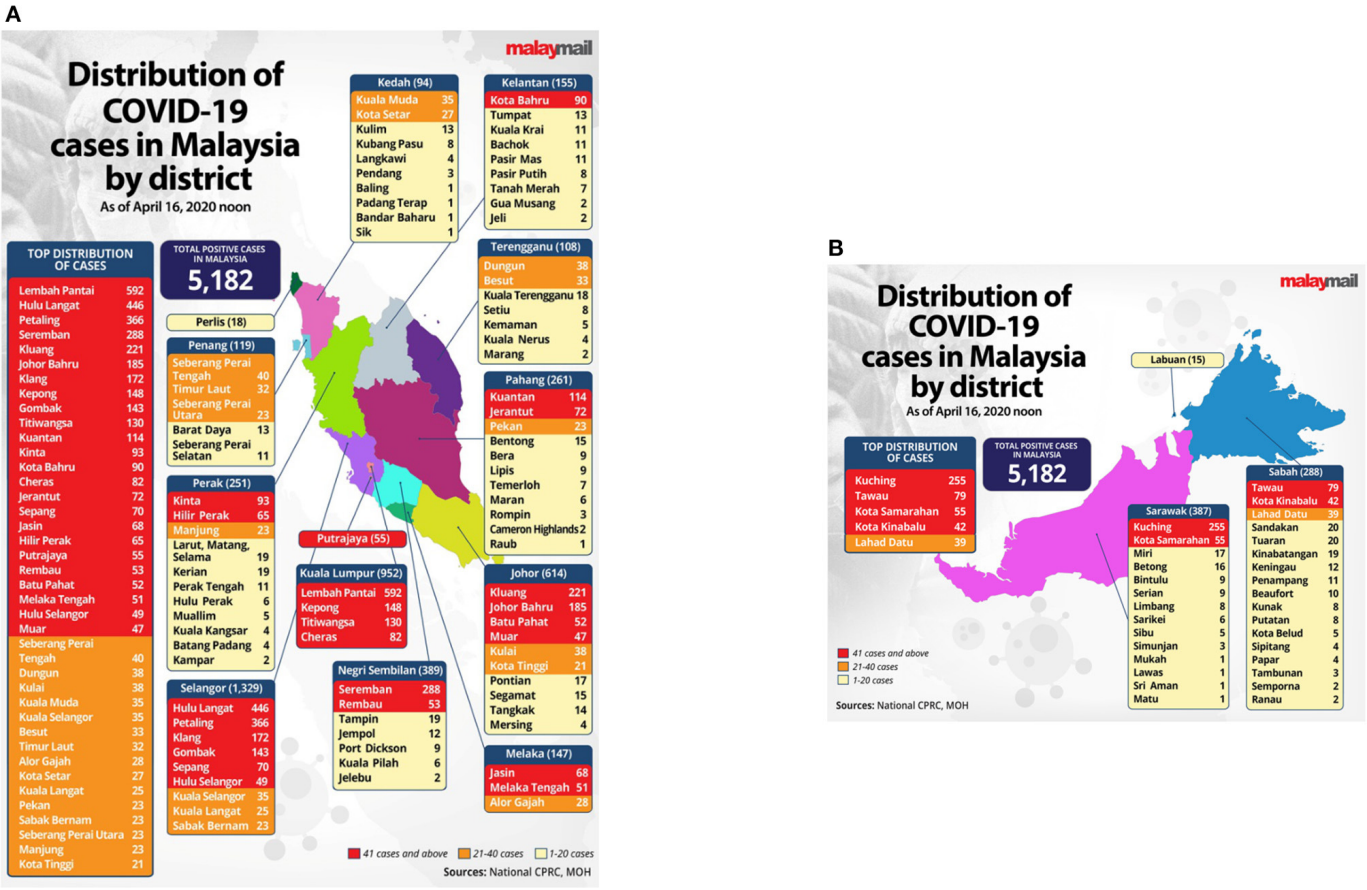
Distribution of COVID-19 cases by district in Malaysia as of April 16, 2020. **(A)** Peninsular Malaysia, **(B)** island of Borneo, Malaysia (10). Source: Malay Mail. Permission has been obtained from the copyright holders.

**Figure 3:**
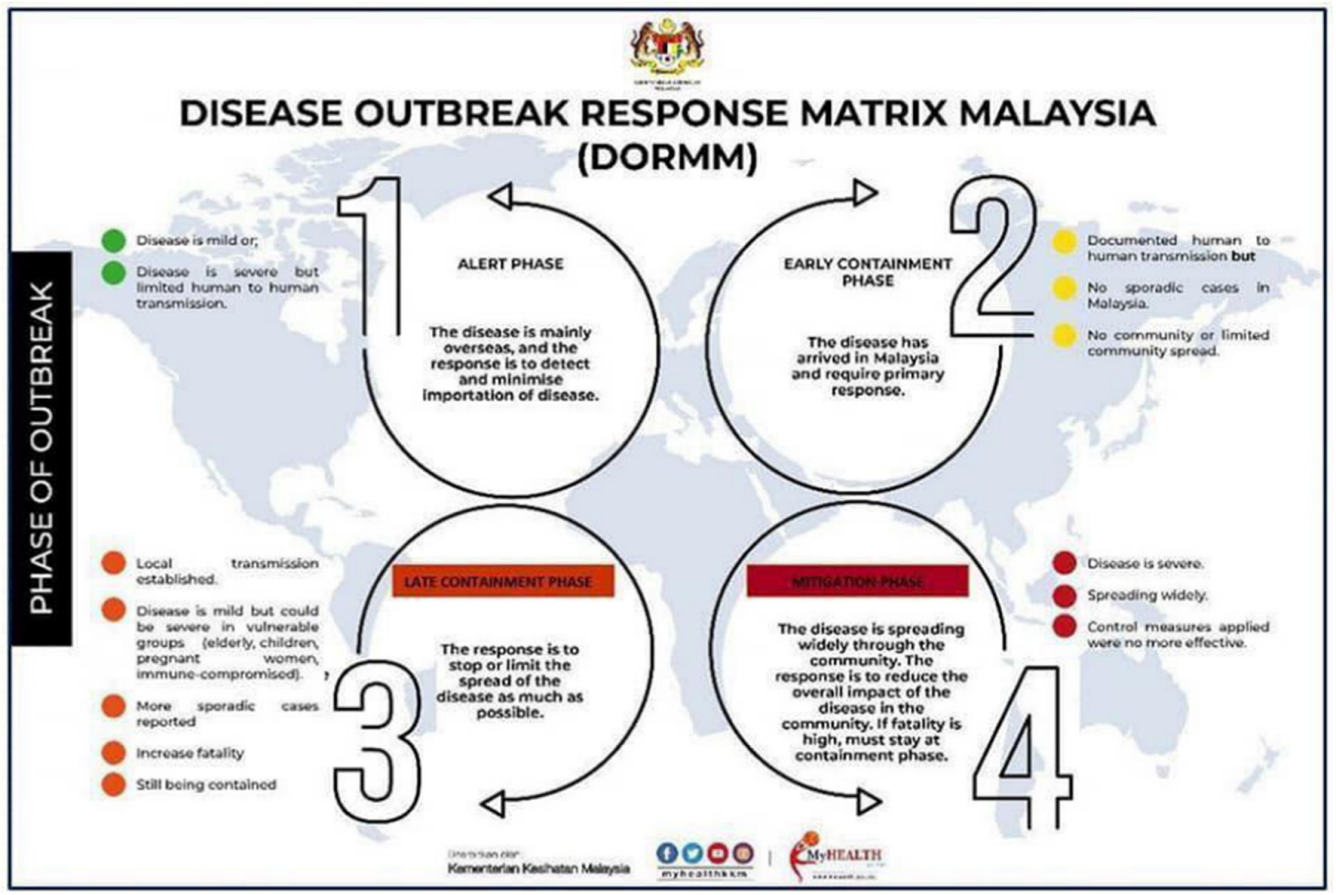
Disease outbreak response matrix Malaysia (11). Source: Ministry of Health, Malaysia. Permission has been obtained from the copyright holders.

Sri Petaling Tabligh became the largest cluster of COVID-19 infection that triggered local transmission across all states in Malaysia (Figure 2) (10). The Ministry of Health (MOH) Malaysia immediately urged all tabligh attendees to contact the local district health offices for screening and risk assessment (11). On March 15, the number of daily new cases surged from 41 to 190 cases from across all states, with most of them linked to the Sri Petaling Tabligh cluster. As the number of new cases continued to exceed 100, and totaling 553 cases on the next day, MOH Malaysia announced the country to be in the late containment phase of COVID-19 (Figure 3) (11), where prompt responses were necessary to stop the disease spread. Later, Malaysia announced the Movement Control Order (MCO), commencing on March 18 to contain the virus through social distancing strategy. On March 19, Malaysia recorded a total of 900 cases, which ranked Malaysia as the country with the fourth highest number of cases in Asia, and the first in Southeast Asia (12).

The Sri Petaling Tabligh cluster generated a number of subclusters especially at Islamic educational institutions (*madrasah*) in several states. Some of the significant subclusters (cases as of April 29) were at Sungai Lui in Hulu Langat, Selangor (157 cases); Sendayan, Negeri Sembilan (81 cases, 1 death); Jerantut, Pahang (81 cases); Rembau, Negeri Sembilan (53 cases); and Jasin, Melaka (40 cases) (13). A number of other tabligh clusters were also identified, namely, the Makasar Tabligh Johor (27 cases, 1 death), Makasar Tabligh Sabah (9 cases), and Pakistan Tabligh Sabah (6 cases) (13). Screening activity from targeted approach among madrasah and tahfiz schools reported 342 cases (5.5%) out of 6,229 students, teachers, and staff screened (14). A wedding reception in Bangi, Selangor was also connected with the cluster, reporting 96 cases which included a number of healthcare workers. By March 19, MOH managed to trace 10,650 of the Sri Petaling Tabligh attendees and detected 513 positive cases in the cluster (15).

As of April 29, a total of 33,577 people had been examined, and 32,590 samples had been taken from the Sri Petaling Tabligh cluster. The cluster reported 2,167 cases which represented 6.5% of total samples taken from the cluster, and 36% of the total 5,945 cases in Malaysia. The number of index cases from the cluster was 767 cases, and up to 5 generations chain of transmission among contacts (family members and friends) were traced in the cluster (16). On May 21, the percentage of cases from the cluster increased to 47% (3,347 cases) out of the total cases (7,059 cases) (17).

The Sri Petaling Cluster came to an end on July 8 (zero active case from the cluster) with the last case being reported on June 11 (18). This cluster represented 38.9% of the total positive cases in Malaysia at the time, with 2,550 (75.6%) Malaysian cases from seven states and 825 (24.4%) non-Malaysian cases from 28 countries. During the 4-month ordeal, a total of 42,023 samples were taken with a positive case rate of 8.03% (3,375 cases), case recovery rate of 98.99% (3,341 cases), case fatality rate of 1.01% (34 cases), and intensive care unit (ICU) treated case rate of 2.58% (87 cases) with 29 cases on ventilators. The number of deaths from this cluster constituted 28.1% of all deaths in Malaysia at the time, where those aged 60–79 recorded the most number of deaths (65%). On the other hand, a total of 2,187 (64.8%) positive cases were asymptomatic.

### Other Clusters and Case Concentrations

Other clusters of positive cases in Malaysia were formed from local mass gatherings and imported cases of PUIs traveling from oversea countries. In Sarawak, three major clusters were registered: a 3-day church gathering in Kuching recorded 176 cases and three deaths; a PUI from Italy recorded infections among 63 cases with five deaths; and a hospital cluster recorded 56 cases, of which 29 cases (52%) were linked to the church gathering (19). In Kuantan, Pahang, a cluster from a PUI with travel history to Bali, Indonesia recorded 43 cases with three deaths, including 10 healthcare workers at a medical center (20). Besides that, a total of 164 imported cases were reported among Malaysians who returned from Indonesia including students (21). From April 3 to 26, a total of 139 cases (1.1%) were detected among 12,672 Malaysians who returned from overseas (14). Among non-Malaysians such as travelers, immigrant workers, and asylum seekers, the MOH detected 601 cases (22), of which most were nationalities from Indonesia and the Philippines.

In relation to clusters, several areas that reported highly concentrated number of cases had been placed under enhanced Movement Control Order (EMCO) to contain the local transmission rates (23). These areas are the Sungai Lui village (156 cases) connected to Sri Petaling Tabligh; Bandar Baru Ibrahim Majid in Kluang, Johor (193 cases, 4 deaths) (24); and Masjid India Road where many foreign immigrants were staying (180 cases) (25). The number of cases at several local wholesale markets in Selayang, Selangor and northern Kuala Lumpur also reported an increase (79 cases), and had been linked to the Sri Petaling Tabligh cluster (26). As wholesale markets are normally visited by large number of people including traders from other markets, the MOH also prompted visitors who believed to have been exposed to come forward for screening, to avoid another huge outbreak such as the Sri Petaling Tabligh event (27).

On the other hand, as healthcare workers (HCWs) carry high risks of infection in direct contact with patients, MOH Malaysia reported that most HCWs got infected outside the healthcare settings and not when handling patients (19). Out of 325 cases (5.8% of total) among HCWs, 70% were infected through community transmission (28).

### Third Wave of Outbreak (October 8–Present)

After lowering to a record of between single- and double-digit number of cases from July to September 2020, Malaysia entered the third wave of outbreak in early October with the highest number of cases coming from Sabah (8,082), Selangor (3,357), Kuala Lumpur (2,853), and Kedah (1,940) from September 1 to October 19 (The Star, 2020) (29). The most significant number of cases was recorded in Sabah, where several large clusters of cases have been identified. These clusters are mainly concentrating in the east of Sabah including Lahad Datu, Semporna, Tawau, and Sandakan area. The largest cluster is the Benteng Lahad Datu cluster at the Lahad Datu District Police Headquarters, which led to several subclusters including the Tawau prison subcluster. The number of cases in Sabah worsened after the Sabah state election on September 26, where persons who returned from high-risk areas in Sabah to peninsular Malaysia were also tested positive (MOH, 2020) (30). At the start of the third wave, the total number of confirmed cases was 14,368 on October 8. In just 2 months by December 3, the number of cases has shot by 381% to 69,095. This also means that 21% of the cases occurred over slightly <10 months during the first and second waves, while 79% of the cases occurred in just 2 months during the third wave (31).

### Infection of Vulnerable Population

**Figure 4:**
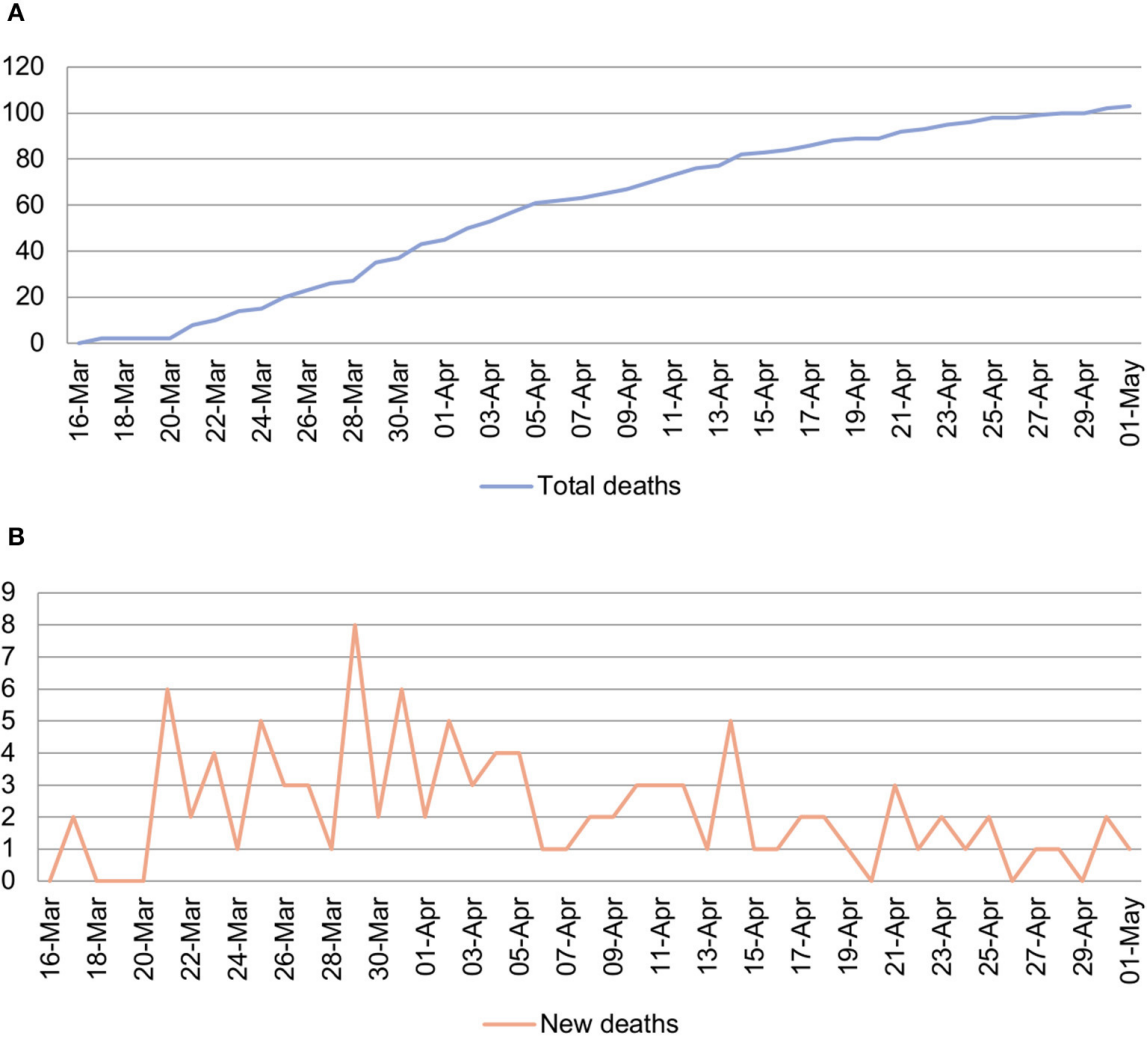
Number of total deaths **(A)** and new deaths **(B)** from March 16 to May 1 in Malaysia. Originally created by the co-author S.C.K.

Similar to the global situation, the elderly and those with chronic diseases in Malaysia were more vulnerable to the COVID-19 infection (32). It was found that the incidence per population rate of COVID-19 cases was the highest among age group 55 to 64 years in the population (33). The first two deaths in Malaysia occurred on March 17 (Figure 4). Among the case fatalities, 63% were reported to be aged above 60 years, and 81% had chronic diseases such as diabetes, hypertension, and heart diseases (34). To protect the vulnerable groups, and at the same time ensure adequate supply of medicine for them without being exposed to high-risk environments such as hospitals, MOH encouraged the use of PharMarchy Value Added Services (VAS) available at MOH healthcare facilities such as medicines through post, drive-through, locker, and appointment system. Besides, a guideline on “Recommendations for the COVID-19 Pandemic for Private, Public and NGO Residential Aged Care Facilities” was also developed especially to manage the elderly care institutions. As of May 1, Malaysia recorded 103 deaths, with a case fatality rate of 1.7% which was lower than the reported rate of 3–4% by WHO (35).

### Detected Mutation of SARS-CoV-2 in Malaysia

One of the notable clusters in the early second wave was the cluster involving the 26th case who had travel history to Shanghai but developed symptoms after a month. As the case had attended several meetings before being aware of the infection, the cluster recorded a total of 121 cases, which was considered as exceptionally infectious compared with the other cases. The Institute for Medical Research later discovered a possible mutation in the virus strain isolated from the 26th case, which might have caused the virus to be more aggressive and contagious (36).

The COVID-19 disease, which is caused by the severe acute respiratory syndrome coronavirus 2 (SARS-CoV-2), has been suggested to be triggered by spike mutation of the SARS-CoV from bats, which enabled it to infect humans (37, 38). Phylogenetic network analysis of the SARS-CoV-2 found the virus in three major variants: strain A which closely resembles that of the bat coronavirus; strain B which is mutated from strain A by a synonymous and a non-synonymous mutations; strain C with a non-synonymous mutation from strain B (39). The main virus strain for the infection in Malaysia is of strain B, which is found exclusively in East Asia. However, it was reported that the other two strains—strain A mostly found in the USA and Australia, and strain C in Europe—were also present in Malaysia (40).

## Threats and Challenges

The origin of COVID-19 outbreak in Malaysia can be traced back to the first case arriving on Malaysian shores on January 25, 2020, when a passenger from China (en route via Singapore) was tested positive for the virus (41). Since then, a historical new chapter unfolded, shaking the pillars of the national public health system and ultimately testing the perseverance of the nation as a whole.

### Disease Containment

The MOH spearheads the national outbreak management of this global pandemic as declared by WHO (42). During the early phase of COVID-19 worldwide spread, even before the first reported case in Malaysia, the MOH has come up with a comprehensive preparedness plan. This plan encompassed several key components including enhanced screening and inter-agency collaborations at entry points (airports, seaports etc.,); bolster sampling at health clinics and hospitals; designation of hospitals and laboratories nationwide as “treating” and “sampling” centers, respectively, empowering the public health surveillance system through active case detection and robust contact tracing; and the adequate stockpiling of personal protective equipment (PPE) and medications needed (43, 44).

The earliest response once the outbreak occurred in Malaysia followed a common standard outbreak management framework emphasizing on thorough case definition formation and case identification process. The MOH distributed the national Guideline on COVID-19 Management aimed in assisting frontliners in every step of management involving COVID-19 cases, including early case detection with clear case definitions used from the outset. The guideline is easily accessible online and has since undergone dynamic editing and updating processes at various points in time as the virus spreads further globally (8). Initially, singular cases were reported almost daily, which in time doubled and later exponentially increased when several clusters mushroomed. As clusters expanded beyond the first generation of contacts, more vigilant contact tracing and testing were done. One notable cluster in Sri Petaling, Kuala Lumpur affiliated to a religious gathering began to surface in early March. Cases began to spread all over Malaysia after the conclusion of the assembly with attendees returning to their hometowns. Moreover, infected international participants later spread the virus to their native countries with cases emerging in neighboring countries like Brunei, Singapore, Cambodia, and Thailand (45). A nationwide call for approximately 16,000 local attendees to come forward for testing ensued (46). Active case detection and mapping of participants led to several mass sampling areas nationwide. Everyone was included in these targeted samplings, from symptomatic to asymptomatic individuals, and both local and foreigners were also not spared; more importantly, tests were carried out by MOH for free. MOH policy clearly stated that no one should be left behind since the virus discriminates no nationality (47). This policy ensures that everyone was treated equally and equitably.

Another method of vigorous case identifications and contact tracing involves targeted active cluster identification. Several areas in Peninsular Malaysia for example recorded sharp case increments justifying localized EMCO to these particular areas. These EMCOs imposed strict no in–out movement, aimed ultimately at reducing and breaking disease transmission. Two such areas in Kuala Lumpur were locations swollen with foreign workers, immigrants, refugees, and asylum seekers thriving with their daily life and businesses, many among them considered marginalized and vulnerable (48, 49). Major samplings of all the residents living in these areas regardless of their nationality were indeed daunting, but consequently led to further detection of cases and their subsequent treatment. Contact tracing has become the mainstay and core activity to curb disease spread and not only confined to regular close family members or contacts, but expanded well beyond that to include shops or places visited by each case. The government also developed and urged the use of technology *via* mobile phone apps aimed to assist COVID-19 outbreak management, and to facilitate contact tracing of people who may be exposed to infected individuals, namely, MySejahtera and MyTrace apps (50). Soon, the three T's of “trace–test–treat” became a new mantra, and this triad was carried out vigorously early on and throughout this ongoing outbreak mainly by MOH staff on the ground (from district and state public health offices) with the help of other agencies, e.g., police, immigration, civil defense personnel, and so on.

Federally imposed isolation and quarantine may be deemed radical by some or even draconian by few, yet these measures (isolation and quarantine) have been two of the most successful tools available in fighting outbreaks since the dawn of epidemics (51). Experience from China showed that these two measures played significant roles in determining the course of COVID-19 and reducing the effective reproduction number (Ro) there (52). In Malaysia, isolation and quarantine of close contacts and travelers returning from abroad were carried out in 409 designated and gazetted quarantine centers nationwide (53). These centers include training centers, technical institutes, community colleges, hotels, and former National Service camps. Quarantine of close contacts and travelers in designated centers were crucial since the MOH estimated that up to 15% of those instructed to self-quarantine at home did not comply with the order (54). In the beginning, these centers were used to house close contacts of positive cases especially among those from the Sri Petaling religious gathering cluster and others from the EMCO areas. Subsequently, as the global spread of the virus outside China worsened, the government brought back Malaysians from all over the world through chartered flights. All of the returnees and close contacts were quarantined for 14 days during which they will undergo viral real-time reverse transcription-PCR (rRT-PCR) sampling twice before they are allowed to return home (if both results returned negative). This “quarantine and test” method later allowed for more case detection among these “travelers” cluster during the later stages of the MCO, subsequently exceeding the number of local infections.

In terms of treatment, the challenge obviously involves selecting a suitable range of evidence-based medications including repurposed drugs to be used in Malaysia. One specific drug that reached stardom and gained renewed popularity for use in COVID-19 treatment is chloroquine (or its more potent derivative hydroxychloroquine). In the COVID-19 management guideline distributed by the MOH, the use of chloroquine and hydroxychloroquine with or without various antiviral medications has been suggested at different clinical stages of the virus (55). These two antimalarial drugs have been suggested to influence the virus–receptor binding of COVID-19 and may impair virus attachment and entry (56). With time, these medications garnered hype worldwide leading to hoarding by several countries despite scanty evidence in regards to their effectiveness (57). However, in light of recent evidences, these repurposed drugs came under heavy scrutiny and was found to have no significant beneficial effect on the treatment and prophylaxis of COVID-19 (58). Malaysia, meanwhile, retracted the use of these off-label drugs in June after analysis showed no effect among 500 COVID-19 patients (59). In the meantime, Malaysia has pledged their commitment with WHO into the global “Solidarity Trial”—an international initiative testing several drugs for the treatment of COVID-19 (60). Nine COVID-19–treating hospitals nationwide were selected to test the efficacy of four treatment regimens using the combination of remdesivir, lopinavir/ritonavir, interferon beta, chloroquine, and hydroxychloroquine (61). WHO recently published their finding on the said trial, dubbed the world's largest randomized clinical trial on COVID-19 therapeutics, and indicated that the regimens had little or no effect on 28-day mortality or the in-hospital course of COVID-19 among hospitalized patients (62).

In the vaccination front, the government looked into several initiatives that may ensure Malaysians will adequately receive successfully developed COVID-19 vaccines from multiple sources worldwide. The government recently pledged their commitment to the global COVID-19 Vaccine Global Access (Covax) partnership, spearheaded by WHO, while also collaborating with the Chinese government, for the procurement of COVID-19 vaccines once they are available (63). A recent concern among many countries including Malaysia is the speed of vaccine procurement. Some Malaysian neighboring countries have started rolling out vaccines procured at rather premium prices because these countries entered into advanced purchase agreements much earlier (before the publication of interim trial data). Singapore, for example, allocated around S$1 billion for vaccine procurements and has since commenced the inoculation of their population (approximately one-fifth of Malaysia's population)—almost the same amount of budget allocated by the Malaysian government for vaccine procurement for our population (64). In a race against time, the government estimated that RM2.05 billion will be used, out of the initial allocated RM3 billion budget, for ongoing vaccine purchase agreements and procurements which involve several pharmaceutical firms worldwide as well as vaccines acquired via Covax. Most of these vaccines require the two-dose regime and will include the Pfizer-BioNTech vaccine, which has obtained conditional approval from the governing National Pharmaceutical Regulatory Agency (NPRA) of MOH, expected to supply 20% or 6.4 million of Malaysia's population (65, 66). It will be rolled out by end of February 2021, during the commencement of the MOH-announced “National Covid-19 Immunization Plan,” outlining a framework of three phases to vaccinate over 80% or 26.5 million of the population to achieve herd immunity over a 1-year period (by February 2022), en route to becoming the largest vaccination program in this country (67). Priority will be given to frontliners in the first phase, followed by the inoculation of vulnerable and high-risk groups (elderly and those with comorbidities), before finally reaching healthy adults aged 18 and above. The Malaysian Prime Minister himself pledged that he will be among the earliest to receive the vaccine (68). The MOH-developed MySejahtera app mentioned earlier will serve as one of the platforms for vaccinees' selection, invitation, enrollment, side effects monitoring, and certification while also reminding vaccinees of their second jab appointments when due (69). All these efforts complement the ongoing research into the most appropriate medications to fight COVID-19 and efficacious vaccines that can provide immunity to this novel virus.

### Threats to the Healthcare System

An important aspect into the ability of nations to combat any new outbreak and one as devastating as COVID-19 is the coping ability of its healthcare system. The whole healthcare system may be stretched thin with improper management and administration. The Malaysian MOH has since the outset prepared for the worst case scenarios and outlined the plan in clear and easily accessible guidelines (8). In times of crisis, the collective collaboration of both public and private healthcare sectors is needed and none should be allowed to work in silos. One such ongoing collaboration is the performance of COVID-19 rRT-PCR tests by certified public and private laboratories (stand-alone or hospital laboratories). As we know, WHO defines a confirmed COVID-19 case as “a person with laboratory confirmation of COVID-19 infection” and the recommended routine testing is through detection of COVID-19 virus RNA by nucleic acid amplification testing (NAAT) such as rRT-PCR (70, 71). In the beginning, as with the government policy to indiscriminately test locals and foreigners during contact tracing, active case detection, and mass sampling, most tests were carried out in government laboratories and they were able to cope with the daily demand of testing. However, with the increasing number of testing per day and increased workload of these laboratories to cope with the turnaround time, private hospitals and laboratories opened their services with significantly reduced fees to share the burden and dependence on public laboratories. Uberization of COVID-tests *via* home sampling was also established with help from the private laboratories. At one point in time, the combined ability of all 43 laboratories (public and private) nationwide reached a maximum of 16,635 rRT-PCR tests per day (72).

By the end of April 2020, the total tests conducted in Malaysia were estimated to exceed 150,000 with the ratio of about 4,700 tests per million population (73). Although, this figure is far lower in comparison with South Korea (11,980 tests per million population) and Singapore (about 17,000 tests per million population), the tests conducted by Malaysia from early on is more of a targeted testing. Tests were carried out undiscriminatingly on symptomatic or asymptomatic local or foreign individuals who were either close contacts (family, workplace, marketplace, school), or those who live in red zone areas, tahfiz (religious) school students, homeless centers, old folks homes, wet markets, construction workers, healthcare workers, returning travelers, and many other risk groups. Meanwhile, the MOH has also approved and received the first batch of antigen rapid testing kit from South Korea which has a reported sensitivity level of 84.4% (74). This effort complements the use of antibody rapid testing kits that have been used to aid the investigation of this ongoing outbreak and to detect seroprevalence of the virus in the community. Surveillance testing has also been conducted nationwide in various sentinel clinics and hospitals for patients who presented with ILI and SARI where out of 6,100 clinical samples collected, 71 (1.16%) were positive for COVID-19 (75). These different testing modalities in the future are hoped to help boost the testing capability and ultimately improve the detection of COVID-19 cases in the country.

Once cases have been tested positive for the virus, the next challenge stems. The adequacy of designated hospital beds along with its ICU beds and ventilators will come into question. The MOH has prepared several contingency plans looking into different best to worst case scenarios. Early on, the government designated 34 public hospitals as the admitting and treating hospitals for COVID-19 nationwide (8). These hospitals were selected based on stringent criteria, among others the number of beds, healthcare staff (specialists, doctors, nurses, etc.,), and adequate infrastructural ability and support system. It later included two other university hospitals into the list. To put into perspective, there are about 150 public hospitals throughout Malaysia (76). In the meantime, large private hospitals have also pledged their readiness for COVID-19 patients if the situation worsens (77). The MOH also introduced “step down” centers where cases who are asymptomatic and clinically stable can be transferred to these centers. This may free up beds in the designated hospitals and reduces the risk of stretching the resources needed. The COVID-19 pandemic has truly challenged the ability of the healthcare system in many countries globally, and Malaysia to some degree experienced the same problem. ICU beds and ventilators are two critical commodities in times of crises. The MCO imposed by the government had generated positive consequences with the number of incidence dropping to two digits toward the end of the third phase of MCO (from April 15 to 28, 2020). Some 40 cases (from the total 1,758 active cases) were receiving treatments in ICU with 18 of them requiring ventilation support (75). As of early May 2020, the health system was able to cope with the demand, with utilization of ventilators standing at around 30% of the total capacity allocated for COVID-19 management (78). The government also allocated a special RM500 million budget to purchase equipment like ventilators and PPE (79).

Other inevitable threats to any healthcare system are the adequacy of manpower and sufficiency of PPE supply. These two must go hand in hand because proper management of COVID-19 requires that each healthcare staff involved must be provided with the appropriate level of PPE. In this time of disaster, PPE are valuable assets hoarded and “hijacked” by some leading to huge demand and inadequate supply to others (80, 81). The scarcity of PPE in certain parts of the world has led to infections and deaths of healthcare staff from COVID-19 infection (82, 83). In Malaysia, the MOH use every possible method of acquiring adequate supplies of PPE for every healthcare staff involved in COVID-19 management (84). Healthcare workers are advised to strictly adhere to guidelines given by the ministry in using the appropriate level of PPEs for different activities they perform during their daily involvement with COVID-19 management. Nevertheless, it is worth acknowledging that many individuals, local entrepreneurs, businesses, and private companies have donated PPEs and even provided monetary funding to buy PPEs. They contributed to ensure that all MOH frontliners are well-protected. Some even went to the extent of voluntarily sewing isolation gowns, head covers, and boot covers while other groups created DIY head covers and donated them to frontliners (85). Everybody is coming together to do their part to help the country battle the COVID-19 pandemic.

Healthcare staff is one of the integral parts in the fight against this virus. As an instrument of the public health system, the healthcare staff serve to protect the health and wellness of the general population. They are the frontliners risking their own safety and health and responsible in activities related to COVID-19. The Ministry mobilizes reinforcements of healthcare workers from states with less COVID-19 cases to states with multiple red zone districts (defined as having 40 active cases or more). MOH has also called on private and retired medical staff to contribute in COVID-19 management nationwide, with a special budget allocated to hire them on contract (79, 86). This is very important to avoid burnout and exhaustion among healthcare workers which could be detrimental to their physical and mental health. The MOH takes this seriously and provided regular tips in avoiding burnouts to all its healthcare staff (87). Many healthcare staff risk being exposed to contracting the infection themselves. Some 325 ministry healthcare staff have been tested positive for COVID-19 so far, and investigations into these cases concluded that none of them contracted the disease in their line of work, with 70% attributable to social gatherings, oversea trips, and others (28). Nevertheless, the guideline provided by MOH clearly outlines the levels of PPE that staff should abide to base on available evidence, which is the responsibility of every staff involved in the COVID-19 management (8). The Health Director-General (DG) has been one of the most respected authorities in the country and his daily national live broadcast ensured a smooth and comprehensive risk communication to all (88). In times of crises, working hand in hand with other agencies ensures a more holistic approach toward achieving the common goal of containing the spread of the virus. These interagency collaborations were carried out in many instances during the mitigation phase when the MCO was initiated and continued throughout.

## Mitigation Strategy

### Movement Control Order

Social quarantine or more popularly known as lockdowns coupled with social distancing has become an almost standard protocol in the control of COVID-19 spread across the world. Lockdown was first implemented in Wuhan, the epidemic epicenter in China, on January 23, 2020. This was soon followed by 15 other cities in Hubei Province, of which Wuhan is its capital, and later by several administrative areas in China. The Wuhan lockdown was only lifted some 2.5 months later on April 8, while in most of Hubei, it was lifted earlier on March 25. Many were concerned of this draconian measure which violated individual rights and were skeptical of its effectiveness. The move, even though commended by WHO, also said that it was beyond its guidelines in epidemic control and was an unprecedented public health measure (89). However, as the pandemic spread to the rest of the world, lockdown implemented in varying degrees became a household term. It is estimated that 1.7 billion or 20% of the world's population have been instructed by their governments to stay home (90).

Malaysia took a similar approach when the number of COVID-19 cases started to escalate during the second wave and implemented the MCO. A phase 1 MCO was first initiated for 2 weeks from March 18 to 31, 2020. This was extended another 2 weeks into the phase 2 MCO from April 1 to 14, and subsequently another 2 weeks into the phase 3 MCO from April 15 to 28 and a further 2 weeks into phase 4 MCO from April 29 to May 12. Thus, the total MCO or lockdown period was intended for 8 weeks. However, from May 4 onwards, the MCO was converted to a conditional MCO (CMCO), with respect to the partial opening of the economic sector as announced by the Prime Minister on his Special Labor Day Address on May 1, 2020. The CMCO continued until June 9, after which the recovery MCO (RMCO) was activated from June 10 to August 31. During RMCO, the economic, education, religious, hospitality, and tourism sectors were reopened, but with strict standard operating procedures (SOPs). These include meetings, conventions, exhibitions, and weddings. The international borders, however, remained closed except for approved travel. On August 28, it was announced that the RMCO was extended until December 31, 2020.

Following the Sabah state election on September 26, 2020, resulting in cases of COVID-19 escalating again with a third wave beginning on October 8, the government reinstated a second CMCO in the state of Sabah on October 13 and in Malaysia's most urbanized area, the Klang Valley comprising Kuala Lumpur, Putrajaya, and Selangor, from October 14 onward. The other states in Peninsular Malaysia, with the exception of Perlis, Kelantan, and Pahang, also joined the Klang Valley in the CMCO on November 9. Under the CMCO, cross-district and cross-state travel were again prohibited unless for work and with prior permission from the police; schools and educational institutions were closed; public events like concerts, conventions, and weddings were again prohibited; religious services were limited to a small group; and public premises like bars and theaters were closed.

The MCO in its various forms was enforced through the Prevention and Control of Infectious Diseases Act 1988, whereby, under Section 11(2) of the Act, the Minister of Health may, by regulations made under this Act, prescribe the measures to be taken to control or prevent the spread of any infectious disease within or from an infected local area (91). Under Section 11(3), an authorized officer may also direct any person or class or category of persons living in an infected local area or in any part thereof to subject himself or themselves to isolation, observation, or surveillance, the period of which is being specified according to circumstances, or to any other measures as the authorized officer considers necessary to control the disease. It is also supplemented by the Police Act 1967 (92).

Subsequently, the Prevention and Control of Infectious Diseases (Measures within the Infected Local Areas) Regulations 2020 was gazetted under the Act by the Minister of Health on March 18, 2020 to facilitate the enforcement of the MCO (91). The MCO incorporated three key measures, namely, implementation of border control, control of public movement, and prohibition of public gathering and promotion of social distancing. Malaysia closed its international border entry points except for foreigners leaving the country and for Malaysians returning from overseas. In terms of movement control, all non-essential work places, commercial establishments, and services were ordered to close down, so that the population nationwide will be confined to their homes and were only allowed to venture out either to perform any official duty, to make a journey to and from any premises providing essential services, to purchase, supply, or deliver food or daily necessities, to seek healthcare or medical services, or for any other special purposes as may be permitted by the Director General of Health. With respect to public gathering, all religious services, wedding receptions, sports events, conferences, cinemas, and public gatherings were disallowed during the MCO period. Malaysians returning from overseas were initially subjected to self-quarantine at home but subsequently they were subjected to mandatory quarantine at government-managed quarantine sites for 14 days. They are tested for COVID-19 on arrival and then again on the 13th day of quarantine. If the result of the second test is negative, they will be allowed to leave quarantine.

There was a slight hitch in the initial implementation of the MCO as it was announced on March 16 to take effect after midnight on March 18. As expected, there were some panicked buying of foods and daily supplies by the population, even though they were told that supermarkets, convenient stores, and restaurants will remain open throughout the MCO. An unexpected event, however, was the exodus of people, especially students from the capital Kuala Lumpur and the surrounding Klang Valley to their homes. Some families also took the opportunity of the 2-week MCO to return to their hometowns across states. This caused congestions at train and bus stations and highways leaving the Klang Valley. Social distancing was ignored, and it is uncertain if the chaos created had resulted in COVID-19 transmission. The government was also worried that the exodus from the Klang Valley might have helped spread the disease to the other parts of Malaysia.

### Enhanced Movement Control Order

**Figure 5:**
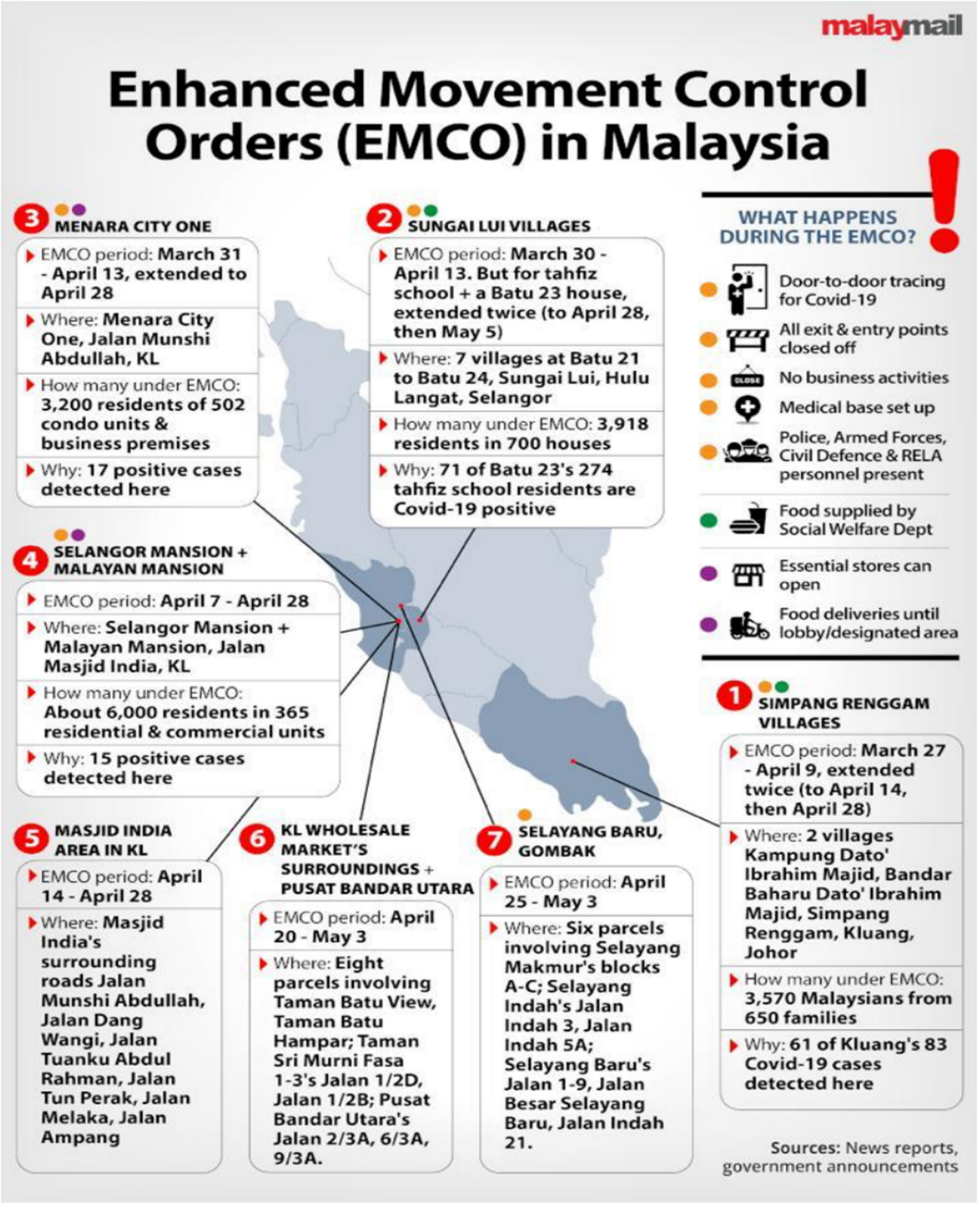
Enhanced movement control order in Malaysia (93). Source: Malay Mail. Permission has been obtained from the copyright holders.

From March 27, specific locations were subjected to a stricter order called the EMCO. Malaysia employed a targeted approach in tackling the COVID-19 epidemic by first identifying high-risk districts and localities. Districts with no active or cumulative case within a 14-day period are termed as green districts, those with 1 to 20 cumulative cases within 14 days are termed as yellow districts, those with 21 to 40 cumulative cases within 14 days are termed as orange districts, while those with more than 40 cumulative cases within 14 days are termed as red or high risk districts. Within each red district, potentially explosive localities are identified and an EMCO may be enforced in those localities. As of April 23, 2020, seven EMCOs were designated, whereby a total lockdown was enforced with the assistance of the police and armed forces. The EMCO can be enforced on a village, a housing area, a commercial area, or an apartment or a condominium (Figure 5) (93). In an EMCO, all residents are required to remain indoor at all times, a medical base is set up, door-to-door screening of all residents for COVID-19 using the RT-PCR method is conducted, and all business activities in the area are ceased except for essential services. Essential food supplies are provided for free to all residents by the Social Welfare Department, all entry and exit points in the area are guarded, and all food deliveries are allowed to deliver only to a designated area. This strategy proved to be very effective in controlling the spread of COVID-19 cases. In a number of these EMCO areas like the Menara City One, Selangor Mansion, Malayan Mansion, Selayang Baru, and residential areas around the Kuala Lumpur Wholesale Market, they are inhabited by foreigners and migrant workers, some of whom are illegals. A number of index cases from these EMCO areas were from the Sri Petaling Tabligh cluster.

### Social Distancing

The Ministry of Health Malaysia defines close contact to a confirmed COVID-19 case as being in social presence of within 1 m from a confirmed case for a duration of not <15 min. Thus, the recommended social distancing for the public is to be apart from each other at a distance of not <1 m. In some countries like the USA and the UK, a social distancing of 2 m is recommended instead. Public Health England (PHE) describes social distancing as steps taken to reduce social interaction between people to reduce the transmission of COVID-19 (94). According to PHE, the objectives of social distancing are more than maintaining the physical distance between persons, which are to

1. Avoid contact with someone who is displaying symptoms of COVID-19 which include a high temperature and a continuous cough.
2. Avoid non-essential use of public transport when possible.
3. Work from home, where and when possible.
4. Avoid large and small gatherings in public spaces like restaurants, leisure centers, and in closed spaces.
5. Avoid gatherings with friends and family while keeping in touch using remote technology such as phone, internet, and social media.
6. Use telephone or online services to contact your general practitioner or other essential services.

### Epidemic Progression

**Figure 6:**
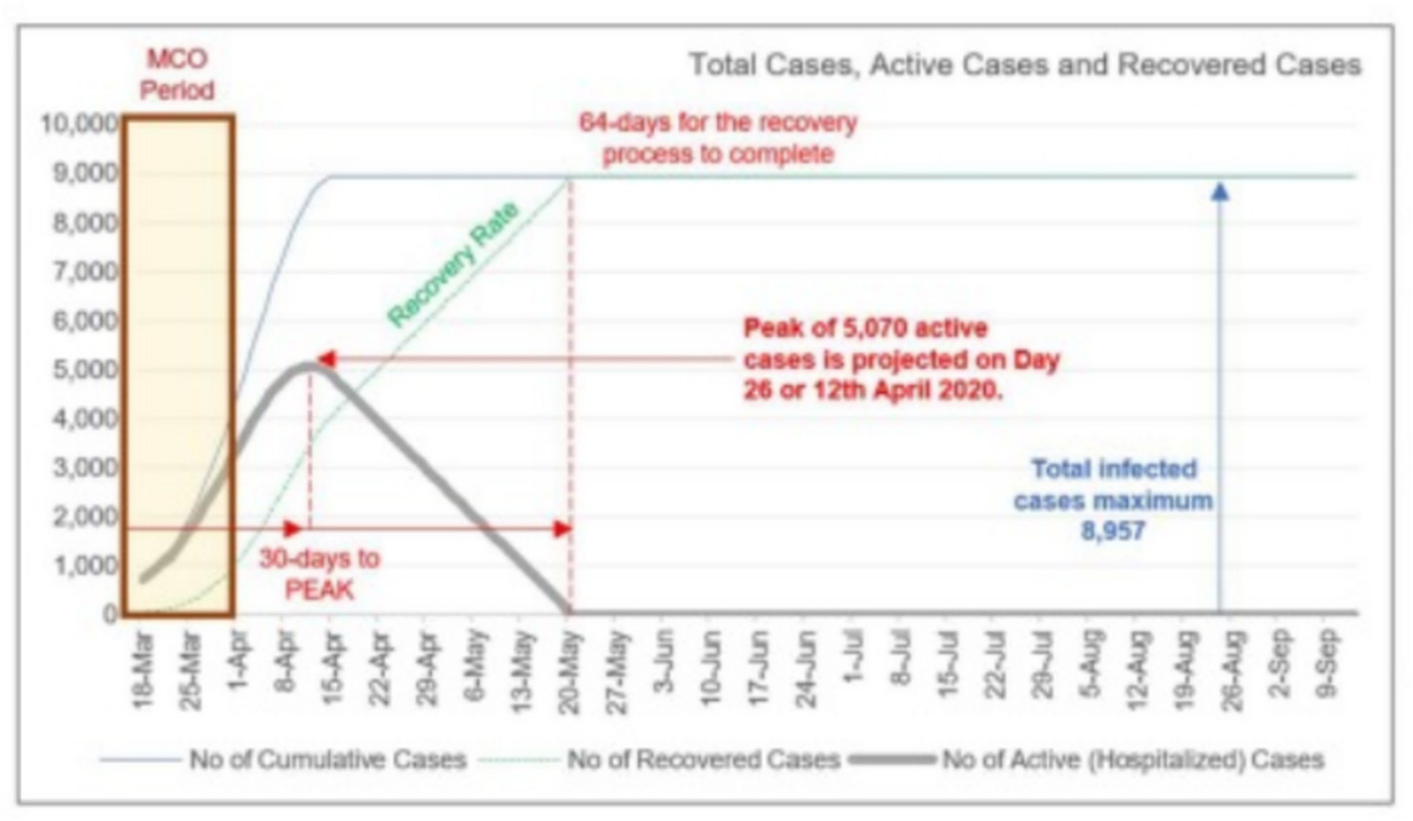
Projection of epidemic peak by the Malaysian Institute of Economic Research (95). Source: Malaysian Institute of Economic Research. Permission has been obtained from the copyright holders.

On March 23, 2020, the Malaysian Institute of Economic Research (MIER) predicted an epidemic peak of 5,070 active COVID-19 cases by April 12, 2020 (Figure 6) (95). This triggered concerns that our healthcare system might be overwhelmed. Thus, efforts were made to increase the number of available hospital beds, for example, the setting up of a temporary medical facility to house mild COVID-19 patients in Serdang, increasing the availability of ICU beds, ordering of medical ventilators, and ordering of personal protective equipment for medical staff.

### Flattening the Epidemic Curve

**Figure 7:**
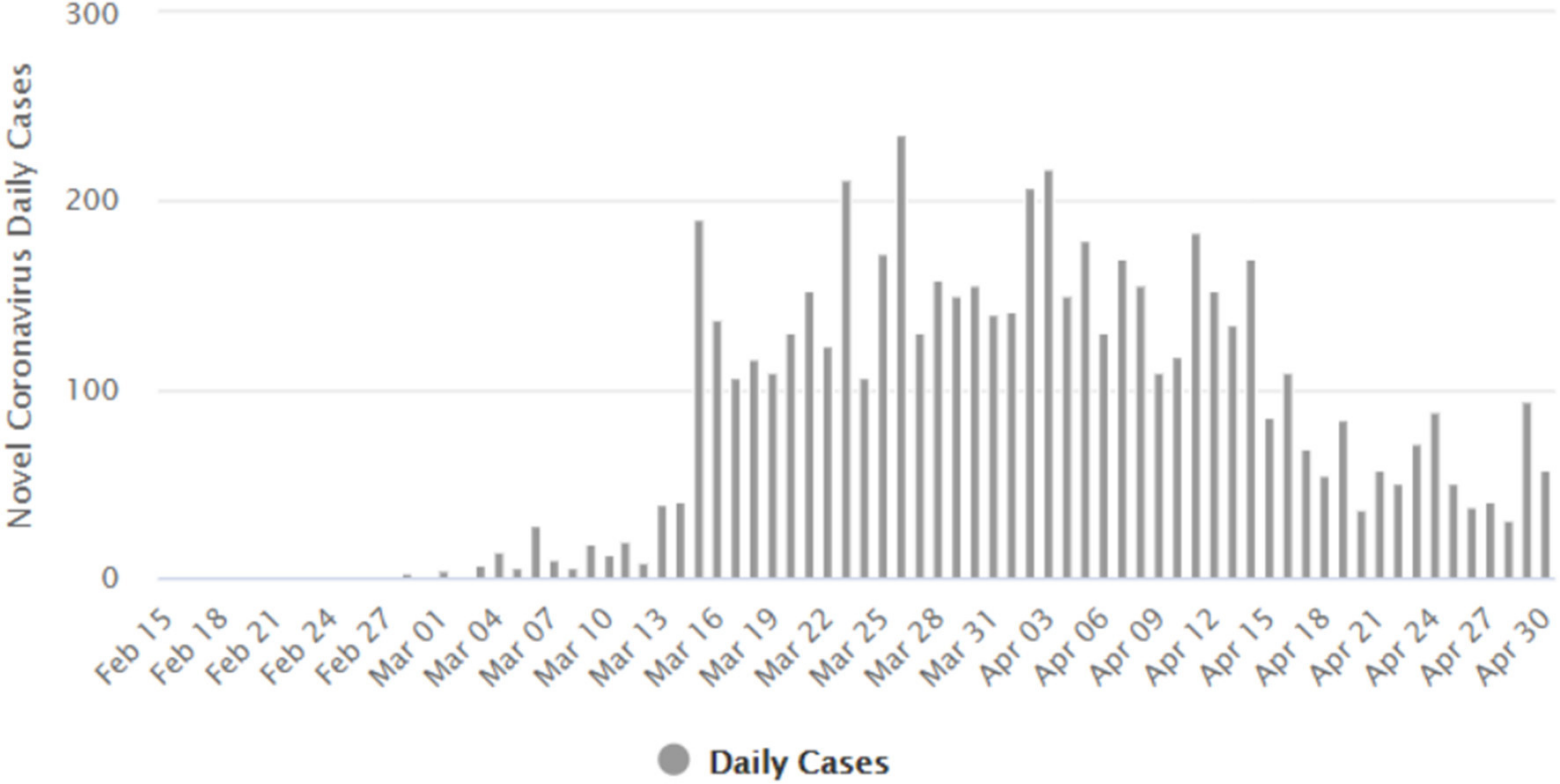
Number of daily new COVID-19 cases reported in Malaysia (96). Source: Worldometers.info. Figure is an open source from the Worldometers.info website.

**Figure 8:**
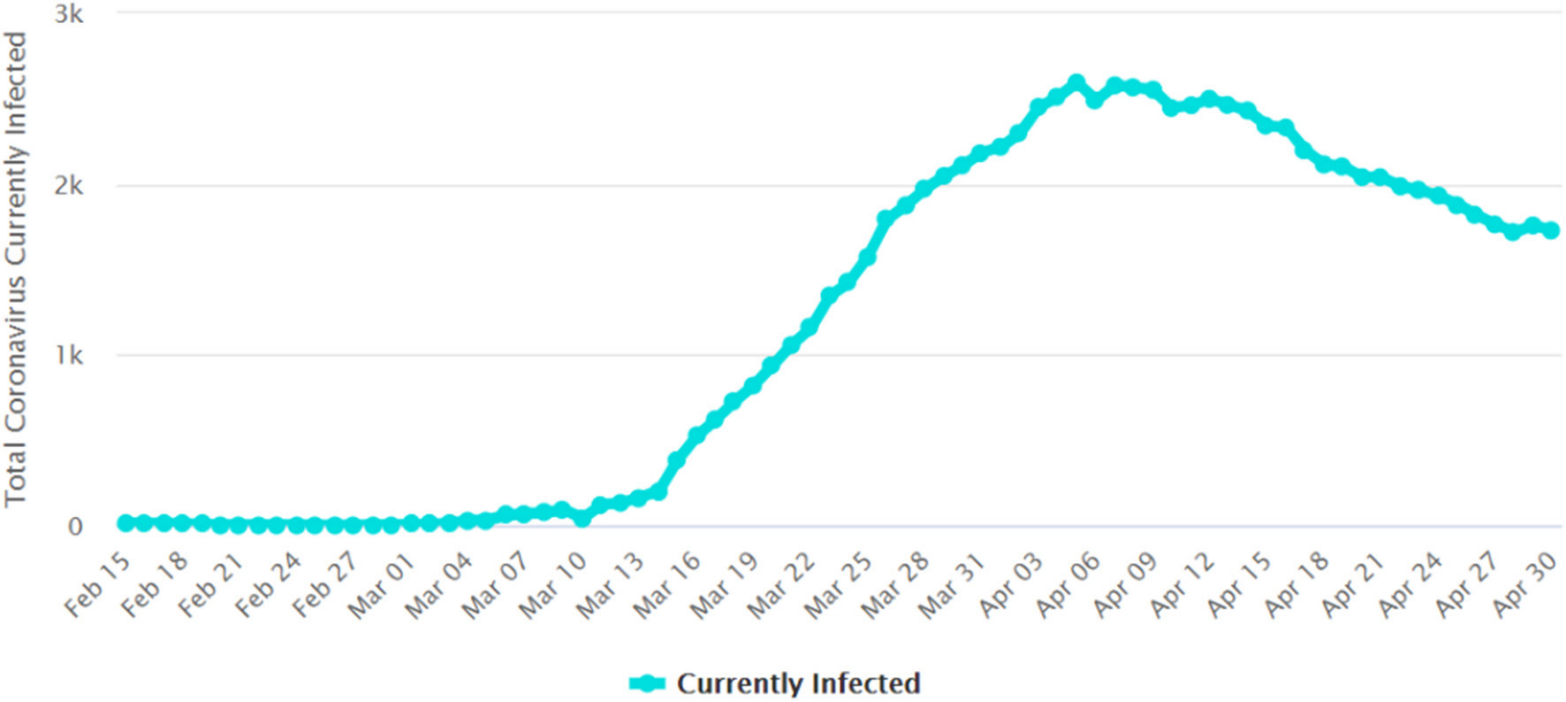
Number of daily active COVID-19 cases reported in Malaysia (96). Source: Worldometers.info. Figure is an open source from the Worldometers.info website.

The implementation of the MCO, especially phases 1, 2, and 3, has clearly managed to flatten the epidemic curve. Figure 7 shows the number of daily new COVID-19 cases reported in Malaysia. The number of new cases peaked at 235 on March 26, 2020 (94). Figure 8 gives the number of daily active cases reported in Malaysia (96). Active cases are cases that are still under treatment in hospitals, which is essentially the cumulative number of COVID-19 cases in Malaysia, minus the total number of recovered cases discharged and the total number of deaths. The highest number of active cases reported was 2,596 cases on April 5. This figure is only 51.2% of the 5,070 cases predicted to occur on April 12 by MIER (95). Therefore, the epidemic curve has been flattened by about half of what it should have been if proactive measures like the MCO were not taken by Malaysia. The epidemic peak also occurred a week earlier on April 5 instead of on April 12 as predicted by MIER.

## Exit Strategy

Many countries in Asia and Europe which have been under lockdowns are now strategizing for an exit strategy to reopen their countries in order to revitalize their economic sector and activities. Austria was one of the earlier European countries to impose a lockdown on March 16, 2020 which has strictly shut down its entire public system and businesses (97). It acted swiftly to close down bars, restaurants, schools, theaters, non-essential shops, and other places of gathering. Austria has since reopened on April 14, 2020 but urged its public to maintain social distancing when in public areas. Three weeks into reopening, it has not seen a new spike in infections (98).

### Reopening the Country

WHO announced six conditions a country must acquire before lifting a lockdown (99). These conditions provide justifications for a country to make a decision to lift restrictions on their social and economic activities:

1. Disease transmission is controlled.
2. Health system capacities are in place to detect, test, isolate, and treat every case and trace every contact.
3. Outbreak risks are minimized in special settings like health facilities and nursing homes.
4. Schools, workplaces, and other essential places have established preventive measures.
5. The risk of importing new cases can be managed.
6. Communities are fully educated, engaged, and empowered to live under a new normal.

However, WHO warned that lifting the lockdown too soon and not carefully managing the opening of a country may lead to a resurgence in new COVID-19 cases and undo all the disease containment efforts that has been painstakingly achieved thus far under lockdown (100).

The MOH has envisaged a soft landing for an exit strategy, in which the social and education sectors may have to wait a longer period and the travel ban may continue after MCO is lifted. In line with WHO's recommendations the MOH felt that the following criteria has been met by Malaysia:

1. The implementation of public movement control to reduce the rate of infection among locals.
2. Upgraded capacity of healthcare facilities.
3. Improved ability to care for population at risk, such as the elderly, treated patients, as well as persons with disabilities.
4. Community empowerment in COVID-19 prevention.
5. Malaysia's border control has been tightened to prevent the importation of positive COVID-19 cases.
6. Adoption of the new-normal practices including social distancing and good personal hygiene.

The undesired impact of the MCO or lockdown is its damaging impacts on a country's economy, which may plunge a country into a recession with widespread unemployment. In a Labor Day address to the nation on May 1, 2020, the Malaysian Prime Minister announced that the country loses about RM2.4 billion a day during the MCO period. The total loss has been estimated at around RM63 billion up to the end of April. If the MCO was to continue for another month in May 2020, Malaysia stands to lose another RM35 billion in revenues, accumulating the loss to RM98 billion (101). This is indeed a significant economic impact on a small developing country like Malaysia with limited economic resources. Thus, Malaysia has to carefully plan an exit strategy that will both help to contain the spread of COVID-19 at a manageable level, but at the same time allow its economic sector to restart.

In facing the current economic crisis amid the COVID-19 pandemic, the Malaysian government adopted a strategy that includes six approaches:

1. A firm action and resolve in controlling the spread of the COVID-19 pandemic by implementing public movement control.
2. Build an economic resilience through a stimulus package known as PRIHATIN to improve people's economy.
3. Regenerate or restart the economy on a structured and controlled basis.
4. Implement economic recovery strategies in facing the new normal.
5. Strengthen or revitalize the economy for future sustainability.
6. Restructure or reform the economic foundation to allow the country and its people to migrate into an era of living with the new normal.

Thus, the Prime Minister also announced a reopening of the country's economic and public sectors on May 4. This essentially converted the fourth MCO into what is called a conditional MCO (CMCO). Under CMCO, there are several categories of industries and businesses that are still not permitted to operate. These businesses or activities involve public gatherings and body contact, whereby social distancing will be difficult to maintain.

Businesses and activities that are still not permitted are movie theaters, karaoke centers, reflexology centers, entertainment centers, nightclubs, theme parks, Ramadhan bazaars, public Ramadhan iftar, Aidil Fitri bazaars, carnival sales, all forms of conferences, and exhibitions, social and cultural events (e.g., weddings, concerts, cultural performances), feasts, open houses, public iftar, monthly gatherings at government and private departments, all forms of council inaugurations and assemblies, religious activities (religious parades, Friday prayers, all activities of worshiping or assembling in mosques, prayer houses, and houses of worship), cross-border travel (except for purpose of attending work and returning home after stranded in villages or elsewhere), and cross-state travel to return to villages for Aidil Fitri holiday. Meanwhile, educational activities in schools, colleges, and institutes of higher learning will continue to be conducted remotely. EMCO in designated areas continued to be enforced. Local public movement are no longer restricted to a 10-km distance but must be within a state. Malaysia's international borders remained closed to entering foreigners and exiting Malaysians.

The MOH has set SOPs for the reopening of the economic sector and businesses starting May 4, 2020 (101). These SOPs will emphasize the following considerations:

1. Social distancing.
2. Personal hygiene.
3. Appropriate use of face mask.
4. Immediate reporting of COVID-19 case to the MOH.
5. Priority in protecting vulnerable population (infants, children, elderly, and handicapped person).
6. Sick persons with symptoms to undergo health screening.
7. Social distancing in public transport.
8. Promotion of online transactions.

### Mass Antibody Testing

Antibody or immunoglobin (IgM and IgG) that are produced in our body's immune system will help to stop foreign viruses from harming our body. Hypothetically, those people who have been exposed to COVID-19 and recovered are expected to develop some level of immunity against the SARS-CoV-2 virus. Therefore, mass antibody testing can be a strategy for countries to detect their population immune response to COVID-19 by specifically looking for antibodies developed against the virus (102).

Several countries mostly in Europe and the USA have started collecting mass antibody data (103). These data would be used as one of the decision-making tools of risk assessment and management for a country to lift its restrictions and open its market. However, WHO has warned countries that there is currently no evidence that people who have recovered from COVID-19 and have antibodies are protected from a second infection. Therefore, this perception of an “immunity passport” should be adopted with precaution (104).

Malaysia has also considered conducting random antibody testing in the red zones areas to know the prevalence of infection in the community, especially among infected persons who have not been detected. This antibody testing helps the government to contain the number of sporadic cases in the country (105).

### Achieving Herd Immunity

Globally, COVID-19 serological datasets from the patients admitted into the hospital with severe symptoms, whereby most of them develop immunoglobulin G (IgG) antibodies of symptomatic infection, correlate with the virus disappearance (106). However, currently there are not enough serological datasets from the non-hospitalized people with positive COVID-19 but without symptoms. A country needs to have mass serological dataset of its community to gauge the status of population immunity toward COVID-19. However, with the current level of natural population exposure to this pandemic, the required level of herd immunity is unlikely to be achieved (107). Meanwhile, experts at Johns Hopkins University warned that mass exposure to the virus in the hope of achieving herd immunity could result in increased mortality which could overwhelm the capacity of a country's healthcare system (108). Malaysia does not want to take the risk of allowing for herd immunity as it was unclear and there is lack of evidence that recovered patients may develop immunity (109). Nevertheless, currently Malaysia has no case of COVID-19 reinfection by recovered patients (110).

### Vaccination

In fighting the global spread of COVID-19, vaccination would be the best approach to acquire herd immunity against the virus. However, in reality, the world's medical experts still have much to learn about this novel virus, and vaccine would only be available within 1 to 1.5 years. While waiting for the arrival of the vaccine, the MOH urge the public to keep practicing social distancing and maintain good personal hygiene.

Malaysia is also calling for cooperation with other vaccine-producing countries to work together in developing the COVID-19 vaccine, and is willing to share her facilities, data, and resources toward this effort. Malaysia is willing to participate in the clinical trials once the vaccine is made available. Malaysia is considered highly suitable for the human vaccine trials, as Malaysia is a multi-racial country (111).

## Conclusion

Even though COVID-19 is a global pandemic, expression of the epidemic may differ from one country to another. This may relate to the virus genetics, vulnerable population characteristics, population's behavior, and the country's response to the crisis. We presented here Malaysia's challenges and response to the epidemic in the hope of sharing our experiences that can be lessons learned for others. Malaysia as a developing country with limited resources faces mounting challenges in responding to the crisis. However, with good cooperation between government agencies and the public, the country managed to overcome the first two waves of the epidemic. Nevertheless, the third wave proved to be more daunting, but is kept under control at the time of writing of this article. Hopefully, Malaysia will be able to keep the epidemic at bay, at least until the arrival of the vaccine.

## Author Contributions

ZH has contributed in writing for part 1 - introduction. SK has contributed in writing for part 2 - COVID-19 epidemic progression in Malaysia. MM has contributed in writing for part 3 - threats and challenges. JH has contributed in writing for the abstract and part 4 - mitigation strategy. MA has contributed in writing for part 5 - exit strategy. All authors contributed to the article and approved the submitted version.

## Conflict of Interest

JH, ZH, and SK were employed by the company Provenue Corporation Sdn Bhd. The remaining authors declare that the research was conducted in the absence of any commercial or financial relationships that could be construed as a potential conflict of interest.
